# Fecal Microbiota in Healthy Subjects Following Omnivore, Vegetarian and Vegan Diets: Culturable Populations and rRNA DGGE Profiling

**DOI:** 10.1371/journal.pone.0128669

**Published:** 2015-06-02

**Authors:** Ilario Ferrocino, Raffaella Di Cagno, Maria De Angelis, Silvia Turroni, Lucia Vannini, Elena Bancalari, Kalliopi Rantsiou, Gianluigi Cardinali, Erasmo Neviani, Luca Cocolin

**Affiliations:** 1 Department of Agricultural, Forest and Food Science, University of Turin, Largo P. Braccini n°2, 10095, Grugliasco, Italy; 2 Department of Soil, Plant and Food Science, University of Bari Aldo Moro, Via Amendola 165/a, 70126, Bari, Italy; 3 Department of Pharmacy and Biotechnology, Alma Mater Studiorum University of Bologna, Via Belmeloro 6, 40126, Bologna, Italy; 4 Department of Agricultural and Food Sciences, viale Fanin 50, 40127, Bologna, Italy; 5 Department of Food Science, University of Parma, Parco Area delle Scienze 49/A, 43124, Parma, Italy; 6 Department of Pharmaceutrical Sciences Università of Perugia, Via Borgo 20 Giugno, 74 06123, Perugia, Italy; Charité, Campus Benjamin Franklin, GERMANY

## Abstract

In this study, the fecal microbiota of 153 healthy volunteers, recruited from four different locations in Italy, has been studied by coupling viable counts, on different microbiological media, with ribosomal RNA Denaturing Gradient Gel Electrophoresis (rRNA-DGGE). The volunteers followed three different diets, namely omnivore, ovo-lacto-vegetarian and vegan. The results obtained from culture-dependent and -independent methods have underlined a high level of similarity of the viable fecal microbiota for the three investigated diets. The rRNA DGGE profiles were very complex and comprised a total number of bands that varied from 67 to 64 for the V3 and V9 regions of the 16S rRNA gene, respectively. Only a few bands were specific in/of all three diets, and the presence of common taxa associated with the dietary habits was found. As far as the viable counts are concerned, the high similarity of the fecal microbiota was once again confirmed, with only a few of the investigated groups showing significant differences. Interestingly, the samples grouped differently, according to the recruitment site, thus highlighting a higher impact of the food consumed by the volunteers in the specific geographical locations than that of the type of diet. Lastly, it should be mentioned that the fecal microbiota DGGE profiles obtained from the DNA were clearly separated from those produced using RNA, thus underlining a difference between the total and viable populations in the fecal samples.

## Introduction

Human intestinal microbiota is a complex ecosystem which contains a huge number of microorganisms, including bacteria, archaea, viruses and fungi, which form 60% of the total fecal mass [1,2]. The composition of intestinal microbiota is gaining importance in human health studies since there is increasing evidence that these microorganisms play a role in disease aetiology [3,4]. Over the last few decades, several studies have reported the importance of improving knowledge on how lifestyle factors, such as diet, age or geographic site, can influence changes in gut microbiota [5–9]. Diet habits in particular appear to be an important factor that affects gut microbiota, in terms of abundance, composition and activity [9–11]. Different kinds of food have been demonstrated to influence microbiota composition, as they provide substrates for bacterial proliferation and function as sources of bacterial contamination. Changing the intakes of the three main macronutrients (carbohydrates, proteins and fats) can significantly affect the composition of microbiota [12–15].

At present, three main dietary habits have been recognized throughout the world: omnivore, ovo-lacto-vegetarian and vegan. The choice of following an ovo-lacto-vegetarian or vegan diet is increasing throughout the world due to the widespread information that is available on the effects that such diets can have on human health, in terms of prevention of cardio-vascular diseases (CVD), cancer and diabetes [16–19]. All these studies indicate that diet has a considerable effect on the composition of gut microbiota. Moreover, comparisons between long-term and short-term dietary habits have shown that only long-term diets are correlated/can be correlated to the composition of microbiota [11].

Although colonic microbiota is relatively stable throughout the life of an adult, age-related changes in the gastrointestinal (GI) tract, as well as changes in diet and immune system reactivity, inevitably affect microbiota [20]. The differences in composition of gut microbiota, related to ovo-lacto-vegetarian, vegan or omnivore diets have been reported extensively, but studies on large cohorts are generally lacking [21–25]. The classical microbiological methods employed for the analysis of fecal microbiota offer a partial representation of the impact on the gut ecosystem, because it has been estimated that more than 60% of fecal bacteria cannot be cultured, due to their fastidious requirements for anaerobiosis and nutritional needs [12]. Molecular techniques, based on the 16S rRNA sequence, such as fluorescent in situ hybridization (FISH), terminal restriction fragment length polymorphism (T-RFLP), denaturing gradient gel electrophoresis (DGGE), quantitative PCR (qPCR) and, more recently, high-throughput sequencing technologies (HTS), are also used to characterize human gut microbiota [11,25,26]. Although DNA based fingerprint procedures provide a picture of the global community, they do not reflect the metabolic activity of the populations, because the DNA could originate from living active cells, living dormant cells, lysed cells, or even dead cells. RNA-based approaches can help overcome this limitation, since they can describe the microbiota, focusing on live bacteria [27]. A clear difference between DNA and RNA based DGGE fingerprints on the predominant fecal bacterial populations has been reported [28,29].

In the present study, the fecal microbiota of 153 healthy volunteers, recruited from different regions in Italy, who followed omnivore, ovo-lacto-vegetarian or vegan diets, has been investigated by means of molecular methods (DNA and RNA-based-DGGE), and using conventional enumeration of the main microbial groups on selective agar plates, with the final goal of describing the impact of the diet regime on the composition of the fecal populations.

## Materials and Methods

### Participant recruitment

Between February 2013 and July 2013, healthy adult volunteers (no = 153) with about equal portions of men and women aged 18–55 (38 ± 9.8), with BMI>18 (22 ± 2.3), who habitually followed an omnivore (total no = 51), ovo-lacto-vegetarian (total no = 51) or vegan (total no = 51) diet, were recruited in 4 different locations in Italy: three locations in the north (Bologna, Parma and Turin) and one in the south (Bari). The volunteers were recruited through advertisements and using flyers, which were distributed in the areas surrounding the collection sites. Additionally, a press release was published on the http://www.scienzavegetariana.it/ web-site.

The subjects had to have followed a specific diet for at least one year at the time of recruitment. An ovo-lacto-vegetarian diet was assumed when the subjects stated they did not consume meat in any form, but ate animal products such as milk, cheese and eggs. A vegan diet was also considered for those who stated they did not consume such animal products. The exclusion criteria were: acute or chronic gastrointestinal diseases; eating disorders, such as anorexia, bulimia or other specified feeding or eating disorder; prevalent chronic diseases, such as diabetes mellitus and cancer; Antibiotic treatment or surgical operations during the previous 3 months; pregnancy and breastfeeding.

### Human fecal sample collection

Volunteers were provided with 3 series of containers to collect the feces (VWR, Milan, Italy); the first contained 10 mL of an Amies liquid transportation medium (Oxoid, Milan, Italy), the second contained 10 mL of RNA later (Life Technologies, Milan, Italy) and the third was empty. The subjects were instructed on how to collect the samples, and all materials were provided in a convenient, refrigerated, specimen collection kit.

The fecal samples were collected at home and transferred to the sterile sampling containers using a polypropylene spoon (3 spoons of about 15g in the Amies containing container, 2 spoons of about 10 g in the one containing RNA later and 2 spoons of about 10 g in the empty one) and immediately stored at 4°C. The specimens were transported to the laboratory within 12 hours of collection at a refrigerated temperature. Each volunteer was requested to collect feces samples over a time span of 3 weeks, once per week. The fecal samples in the Amies based containers were used immediately to evaluate the microbial populations through the use of selective culture media, while the RNA-later based and the empty containers were stored at -80°C for RNA and DNA extraction, respectively.

### Microbiological analysis

Ten g of feces from each volunteer, were homogenized with 90 mL of Ringer’s solution (Oxoid) for 2 min in a stomacher (LAB Blender 400, PBI, Italy; stomacher bags: Sto-circul-bag, PBI, Italy) at room temperature for each week of collection. Decimal dilutions were prepared in quarter-strength Ringer’s solution, and aliquots of 0.1 ml of the appropriate dilutions were spread onto several media, as reported in Table 1. The results were calculated as the means of Log colony forming units (CFU) for three independent determinations.

**Table 1:** Media and cultivation condition used.

Target population	Media	Cultivation condition
Aeromonas_Pseudomonas	GSP agar* plus Pennicillin G** (100 IU/mL)	Aerobic/25°C/48h
*Bacteroides fragilis* group	Bacteroides Bile Esculine Agar°°	Anaerobic/37°C/48h
Bacteroides_Prevotella	Wilkins-Chalgren Anaerobe Agar° plus G-N Anaerobe Supplement° (1 vial/500mL) and defibrinated blood° (25 mL/L)	Anaerobic/37°C/48h
Bifidobacteria	Bifidobacterium Agar°°	Anaerobic/37°C/48h
Coliforms	Chromocult Coliform Agar*	Aerobic/37°C/24h
Corynebacteria	Hoyle medium base° plus Laked Horse Blood° (50mL/L) and Potassium Tellurite° (10ml of 3.5% v/v solution)	Aerobic/37°C/48h
Enterobacteria	MacConkey agar N°2°	Aerobic/37°C/48h
Enterococci	Slanetz and Bartley medium° plus Tween 80** (1 mL/L) and Sodium Carbonate** (40 mL/L)	Aerobic/37°C/48h
Leuconostoc	Rogosa Agar° plus Glacial Acetic acid** (1,32 mL/L)	Aerobic/25°C/72h
Mesophilic_lab	De Man Rogosa and Sharpe agar°	Anaerobic/25°C/72h
Mesophilic_lactobacilli	Rogosa Agar° plus Glacial Acetic Acid** (1,32 mL/L)	Aerobic/30°C/48h
Staphylococci	Mannitol Salt Agar° plus Egg Yolk solution° (10% v/v)	Aerobic/37°C/36h
Streptococci_Lattococci	M17 agar° plus Glucose° (100 mL/L of glucose solution 10%v/v)	Aerobic/37°C/48h
Thermophilic_lab	De Man Rogosa and Sharpe agar°	Anaerobic/42°C/48h
Thermophilic_lactobacilli	Rogosa Agar° plus Glacial Acetic Acid** (1,32 mL/L)	Anaerobic/42°C/48h
Total_anaerobic	Wilkins-Chalgren Anaerobe Agar° plus defibrinated blood° (25 mL/L)	Anaerobic/37°C/48h

Suppliers:

*Merk-Millipore, Darmstadt, Germany;

**Sigma, Milan, Italy;

°Oxoid, Milan, Italy;

°°Becton Dickinson, Milan, Italy.

### Total DNA and RNA extraction from feces samples

The 3 aliquots of feces collected during the experiment from each volunteer were pooled together for nucleic acid extraction. Ten g of the pool was aseptically homogenized with 90 ml of Ringer’s solution (Oxoid) for 2 min in a stomacher (PBI) at room temperature for DNA extraction. An aliquot of two ml was collected and centrifuged at the maximum speed for 30 s, then the supernatant was removed and the DNA was extracted from the pellet using a Powersoil DNA kit (MO-BIO, Carlsbad, CA, USA), according to the manufacturer’s instructions. DNA was quantified using a NanoDrop 1000 spectrophotometer (Thermo Scientific, Milano, Italy) and standardized at 50 ngμl^-1^. About 250 mg of the feces pool was directly subjected to RNA extraction using a Stool Total RNA Purification Kit (Norgen Biotek Corp. Ontario, Canada), according to the supplier's instructions. Seven μl of TURBO-DNase (Life Technologies) was added to digest the DNA in the RNA samples, with an incubation of 3 h at 37°C. RNA was quantified, as described above, and standardized at 500 ngμl^-1^. Each RNA solution was checked for the presence of residual DNA by performing PCR amplification. When positive signals were detected, the DNase treatment was repeated.

### Reverse transcription

Reverse transcription (RT) reactions were performed using M-MLV reverse transcriptase (Promega, Milan, Italy). Five hundred ng of RNA was mixed with 1 μl of 10 μM Random Primers (Promega) and DNase- and RNase-free sterile water (Sigma) to a final volume of 10 μl and then incubated at 75°C for 5 min. The mix was placed on ice and a mixture containing 50 mM Tris-HCl (pH 8.3), 75 mM KCl, 3 mM MgCl2,10 mM DTT, 2 mM of each dNTP, 1 μl of 200 Uμl^-1^ M-MLV and 0.96 U of RNasin ribonuclease inhibitor (Promega) was transferred to the reaction tube. Reverse transcription was carried out at 42°C for 1 h.

### PCR amplification and DGGE analysis

One μl of DNA, or cDNA, was used as a template in the PCR reaction. Two regions of 16S rRNA were amplified: the V3 region with the 338f-GC/518r primers [30] and the V9 region with the Ec1392-GC/Ec1055 primers [31]. PCR template was denatured for 5 min at 94°C, the initial annealing temperature was 66°C and this was decreased 1°C every cycle for 10 cycles, finally 20 cycles were performed at 56°C. The extension for each cycle was carried out at 72°C for 3 min while the final extension was at 72°C for 30 min. PCR products obtained with the two couples of primers were applied to an 8% (wt/vol) polyacrylamide gel (acrylamide- bis acrylamide 37:5:1) with a denaturing gradient ranging from 25 to 55%. The gels were subjected to a voltage of 200 V for 4 h at 60°C, stained for 30 min in 1X TAE containing 1X SYBR gold (Life Technologies) and then analyzed under UV using UVI pro platinum 1.1 Gel Software (Eppendorf, Hamburg, Germany). Selected DGGE bands, specific of each dietary habit, were excised from the gel with sterile pipette tips and purified in water. One microliter of the eluted DNA was used for the re-amplification using the primers and the conditions described above, and the PCR products were checked by means of DGGE. The original PCR product was run on the gel as the control. Products that migrated as a single band and at the same position as the control were purified using a PCR Extract Mini Kit (5PRIME, Hilden, Germany), according to the manufacturer’s instructions. Sequencing was performed with a Deoxy terminator cycle sequencing kit (Perkin-Elmer Applied Biosystems), using the 518r or EC1055 primers. Searches were performed in public data libraries (GenBank) with the Blast search program (http://www.ncbi.nlm.nih.gov/blast/) in order to determine the closest known relatives of the obtained partial 16S rRNA gene sequences.

### Statistical analysis

Data obtained from the plate counts were analyzed using one-way analysis of variance (ANOVA) for each individual volunteer, with diet being the main factor, and the R package “Vegan” (www.r-project.org). ANOVA analyses were performed with the SPSS 22.0 statistical software package (SPSS Inc., Cary, NC, USA). The Duncan HSD test was applied when ANOVA revealed significant differences (P < 0.05). A database of fingerprints was created using the Bionumerics software, version 4.6 (Applied Maths, Sint Marten Latem, Belgium). A combined data matrix that included all the fingerprints of both couples of primers was obtained, and dendrograms of similarity were retrieved using the Dice coefficient and the Unweighted Pair Group Method with the Arithmetic average (UPGMA) clustering algorithm [32]. The similarity distance matrix generated through the Bionumerics software was used to build a Projection on Latent Structures—Discriminant Analysis (PLS-DA) utilizing the “mixOmics” R package. Binary band-matching tables, obtained by means of Bionumerics software, were considered to calculate the Shannon-Wiener diversity index (*H’*) using PAST (PAleontological STatistics) software [33].

### Ethics statement

During a preparatory consultation interview, the candidates were informed about the scope of the research; all the subjects gave written informed consent, and the study was approved by the Ethics Committee of (i) Azienda Sanitaria Locale Bari (protocol No.1050), (ii) Azienda Ospedaliera Universitaria of Bologna (protocol No. 0018396), (iii) Province of Parma (protocol No. 22884) and (iv) the University of Torino (protocol No.1/2013/C).

## Results

### Plate counts

**Fig 1:**
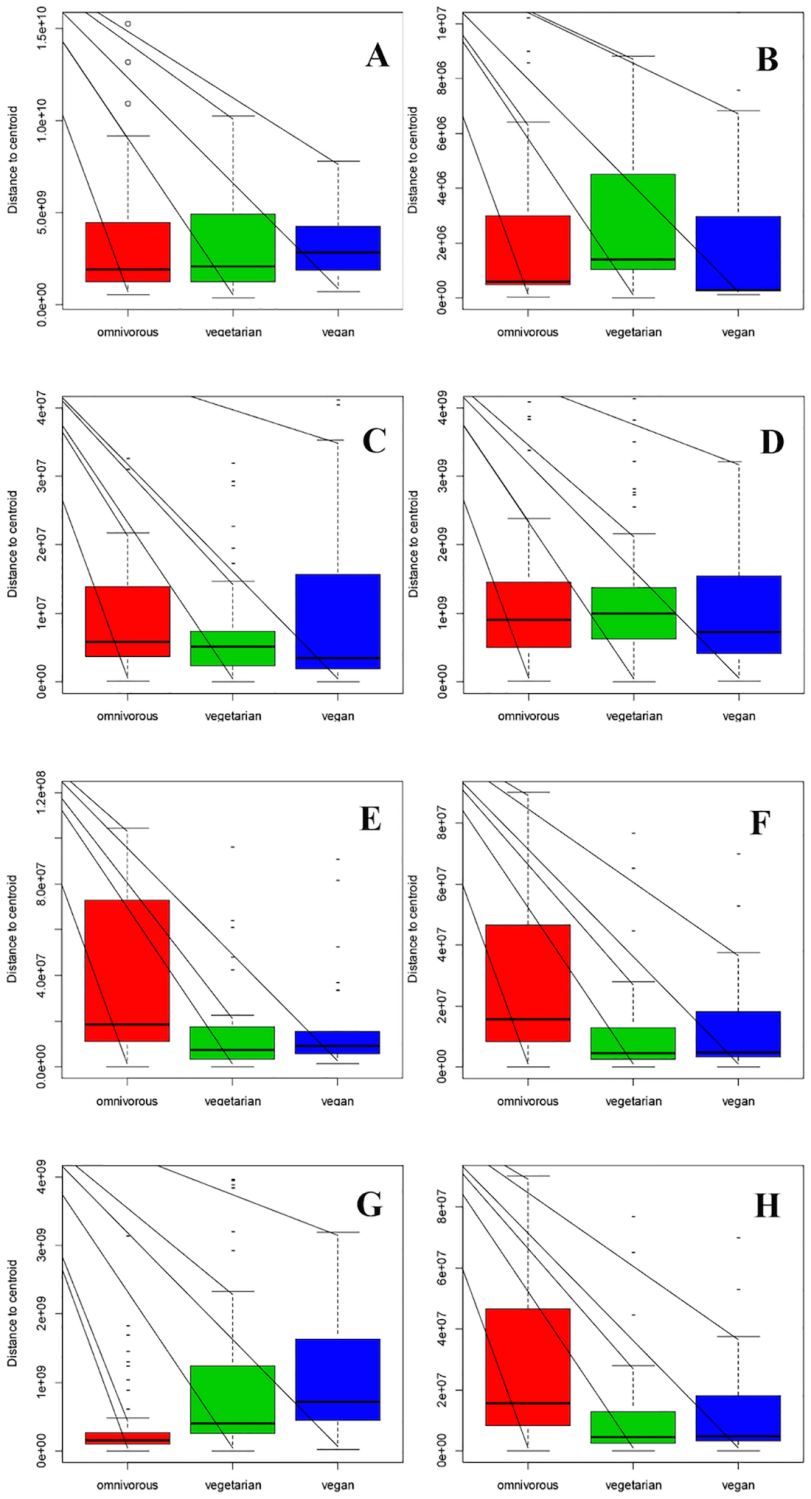
Distribution of the viable counts of the three diets according to the different culture media. Box plot interpretation: the central line indicates the median, upper and lower box lines of the first and third quartiles (Q1 and Q3), respectively, and the bars at the end of the whiskers represent the distribution extremities, while the dots indicate outliers. Plot A, all the media considered together; Plot B, GSP medium at 25°C; Plot C, Chromocult Coliform Agar at 37°C; Plot D, Bifidumbacterium Agar at 37°C; Plot E, MRS at 25°C; Plot F, MRS at 37°C; Plot G, Wilkins-Chalgren Anaerobe Agar + GN Supplement; Plot H, Bacteroides Bile Esculine Agar. For specifications on the counted microbial groups, reference can be made to Table 1.

The results of the viable counts were all considered to calculate a data matrix, which was then processed by PLS-DA. Samples in the 3D dispersion picture (S1 Fig) may appear all closely grouped, but it was possible to find a certain degree of separation of the omnivore from the non-omnivore subjects. The viable count data were further analyzed using ANOVA, with diet being the main factor. The Duncan HSD test was applied when ANOVA revealed significant differences (P < 0.05). Only the results considered significant according to ANOVA were used to build the box plot (Fig 1). No significant discrimination was obtained when all the media were considered (Fig 1A). In the vegetarian group, only the counts on GSP agar (for detection of *Pseudomonas* sp. and *Aeromonas* sp.) showed significant differences (P < 0.05) when compared to omnivore and vegan groups, and they were approximately 1 Log higher than the other dietary groups (Fig 1B). Moreover, the Coliforms (Fig 1C) and *Bifidobacterium* sp. (Fig 1D) counts were lower in the vegan group than in the other two groups (P < 0.05). In addition, the microbial populations counted on MRS agar at 25°C and 37°C showed a remarkable similarity between the vegan and ovo-lacto-vegetarian diets, and a remarkable difference from the omnivors (Fig 1E and 1F). On the other hand, the Bacteroides and Prevotella load, which was monitored on Wilkins-Chalgren Anaerobe Agar plus G-N Anaerobe Supplement, showed a significant reduction (P < 0.05) of about 1 Log in the omnivore group, compared to the vegans and ovo-lacto-vegetarians (Fig 1G).

The population counts of the *Bacteroides fragilis* group, (Fig 1H) in the omnivore group were higher than those of the other groups (P < 0.05).

### DGGE analysis of the fecal microbiota

**Fig 2:**
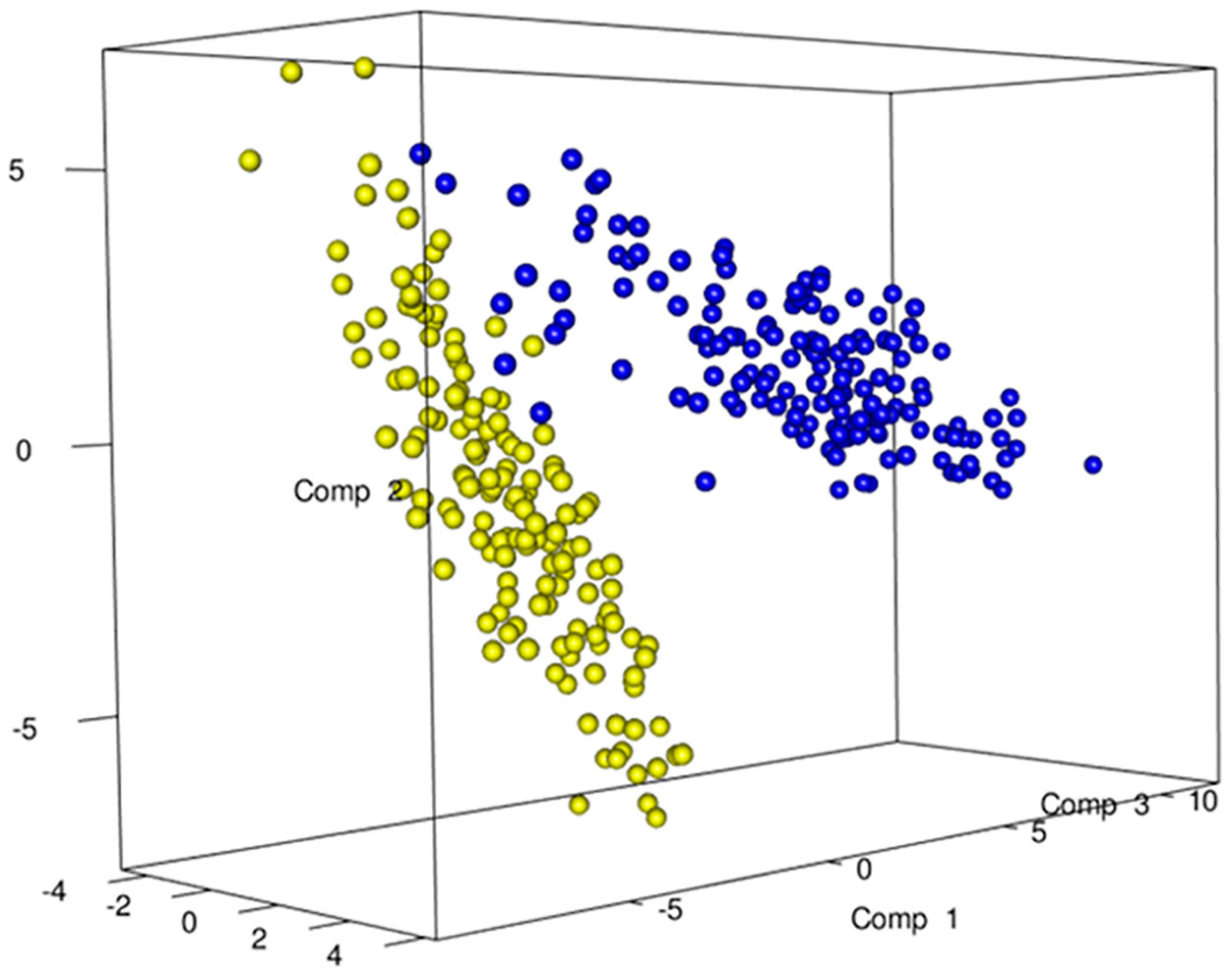
PLS-DA models based on the similarity distance matrix of DGGE built as a function of the nucleic acids. Data from DNA (blue) and RNA (yellow).

**Fig 3:**
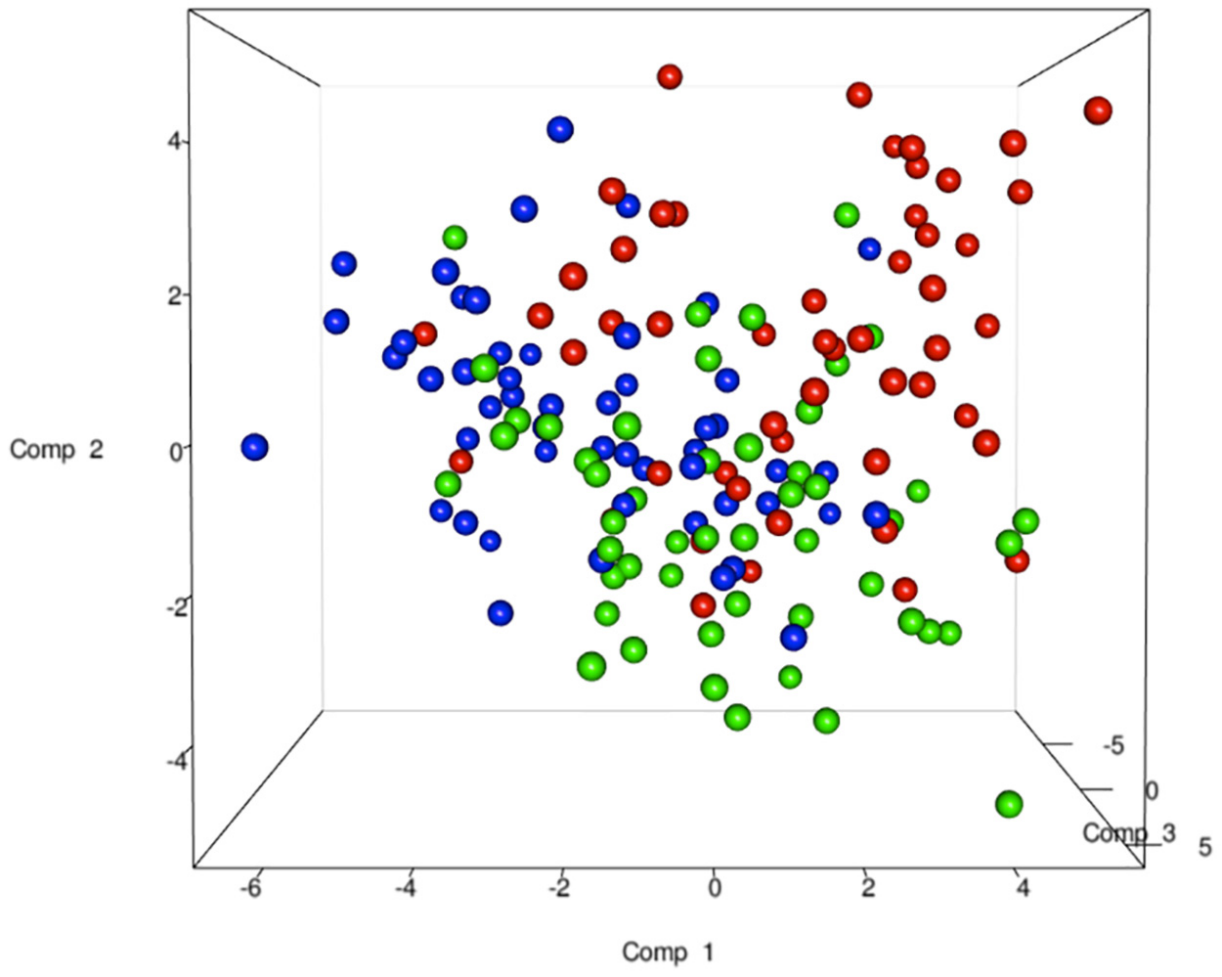
PLS-DA models based on the RNA-DGGE similarity distance matrix as a function of the diet. Data from omnivore (red), ovo-lacto-vegetarian (green) and vegan (blue) individuals.

**Fig 4:**
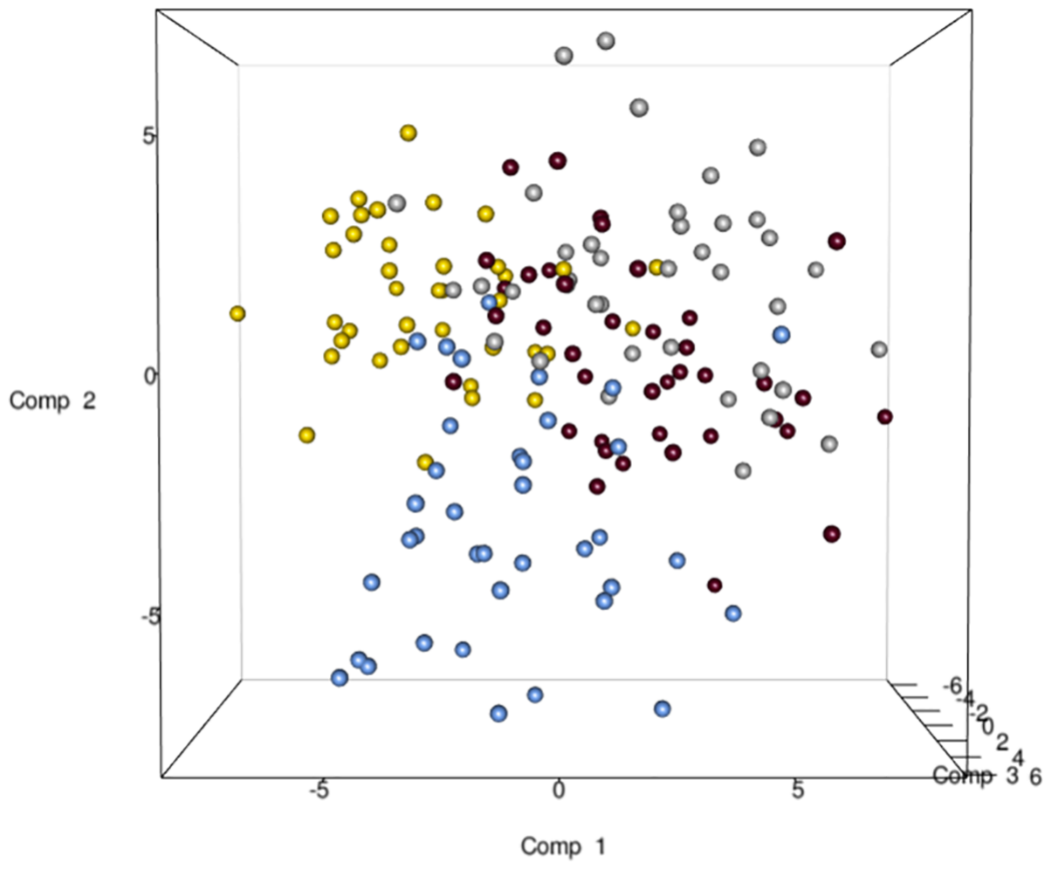
PLS-DA models based on the RNA-DGGE similarity distance matrix as a function of the geographical site. The plot is color coded as a function of the city: Bari (silver), Bologna (gold), Parma (blue), Torino (maroon).

**Fig 5:**
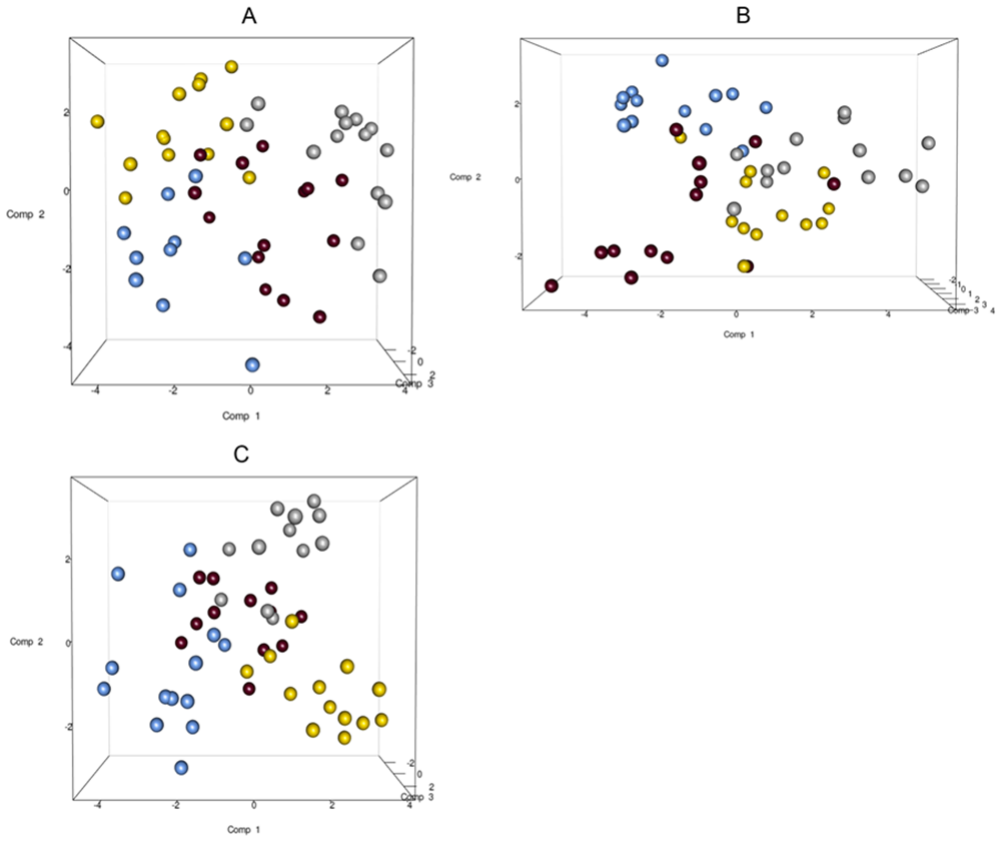
PLS-DA models based on the RNA-DGGE similarity distance matrix from each diet group using the geographical site as the discriminating factor. Plot A, omnivore individuals; Plot B, ovo-lacto-vegetarian individuals; Plot C, vegan individuals. The samples are color coded according to the geographical site (Bari: silver; Bologna: gold; Parma: blue; Torino: maroon).

The total DNA and RNA extracted directly from the feces were employed to amplify the V3 and V9 regions of the 16S rRNA genes, and PCR products of approximately 250 bp, which were analyzed by means of DGGE, were obtained. The DNA based DGGE analysis of the V3 region on average resulted in 22.5 bands per sample (min 7, max 31), while 14 bands per sample (min 3, max 25) (S2 Fig) were obtained for the V9 region. For the RNA based DGGE analysis, an average of 9.8 (min 3, max 18) and 7.4 (min 2, max 22) bands per sample was observed for the V3 and V9 regions, respectively (S3 Fig). A combined data matrix of the fingerprints was produced using the two couples of primers. The similarity matrix generated through the Bionumerics software for the DNA and RNA samples was used to build a PLS-DA, as a function of the nucleic acids, and the results showed a clear separation between the RNA and DNA samples (Fig 2). As far as the dietary habits are concerned, it was only by using RNA data that it was possible to observe a gradient of samples which indicated a certain degree of separation of the omnivore from the non-omnivore subjects (Fig 3), and for this reason, the DGGE profiles obtained from the RNA were subsequently considered. Analyzing the data from each geographical site separately, no differentiation was found between the three types of diet, although some separation was observed between diets in the samples from Torino (S4 Fig). Regardless of the dietary habits, the PLS models showed a separation of samples between Parma and the other recruitment sites (Fig 4). When the samples were grouped on the basis of dietary habits, the PLS-DA models showed a trend of differentiation according to the geographical origin of the samples in the omnivore and vegan subjects (at least for Bari, Parma and Bologna), while the samples from Torino were only clearly separated in the case of the ovo-lacto-vegetarian population (Fig 5).

A binary band-matching table (for the V3 and V9 fingerprints) was analyzed in order to calculate the index of diversity (Shannon-Wiener diversity index *H’*). The *H’* index was further analyzed using ANOVA, with diet or geographical site being the main factors. The Tukey HSD test was applied when ANOVA revealed significant differences (P < 0.05). The results, using only the data from the V3 region, revealed, on the basis of their geographical origin, that there was a significant biological diversity in the samples. In particular, the Parma group showed a significant reduction in diversity *H’* (P < 0.05) compared to the other groups. Moreover, Bari and Torino showed the highest diversity *H’* (P < 0.05). No difference was found using diet as the main factor.

**Fig 6:**
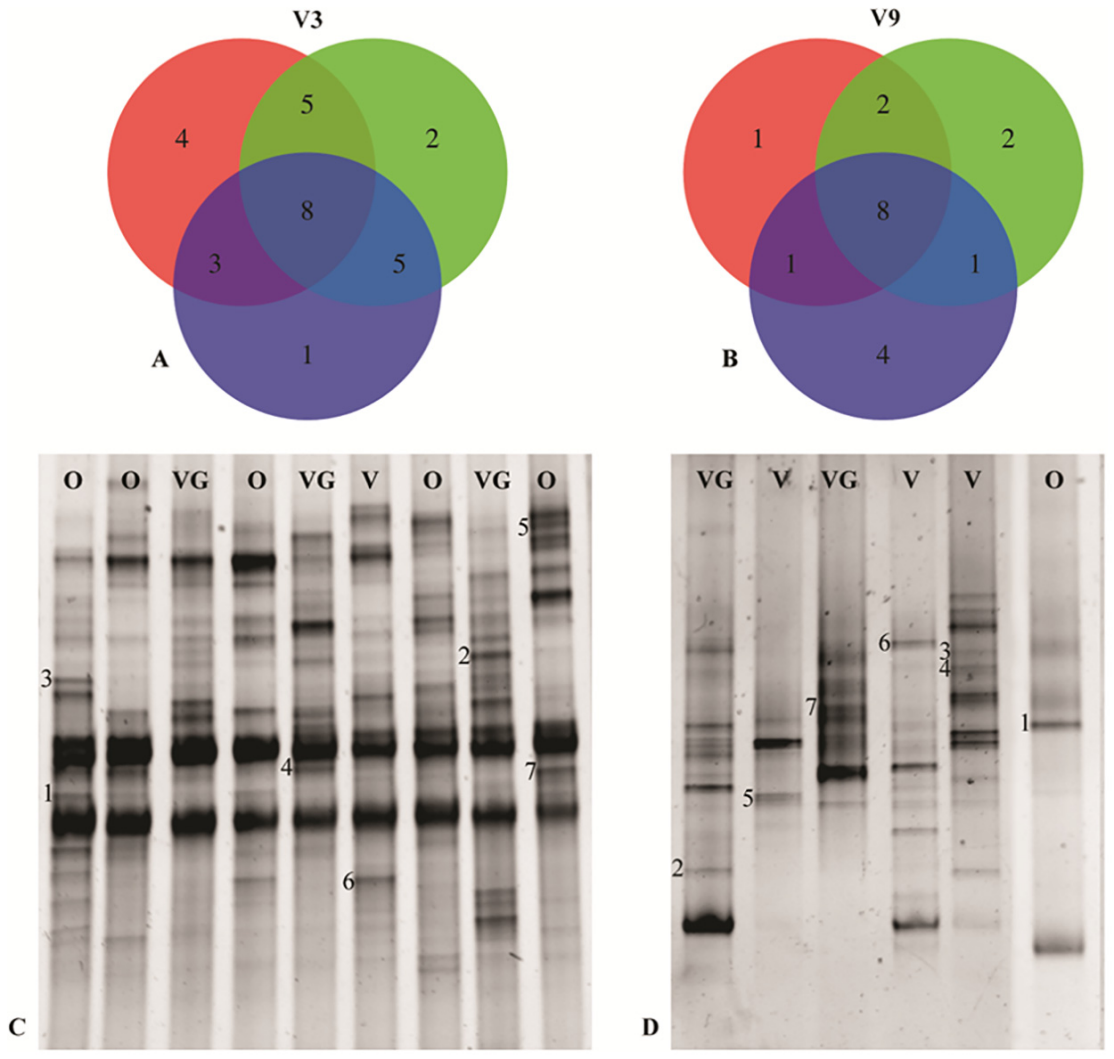
RNA-DGGE band distribution. Plot A and Plot B, Venn diagrams of the unique and overlapping bands present in the RNA-DGGE profile of the omnivore (red), ovo-lacto-vegetarian (green) and vegan (blue) individuals for the V3 and V9 regions, respectively; Plot C, RNA-DGGE fingerprints of the V3 amplicons, where the letters indicate the dietary groups: the omnivore (O), ovo-lacto-vegetarian (VG) and vegan (V) band identifications are reported in Table 2; Plot D, RNA-DGGE fingerprints of the V9 amplicons; the band identifications are reported in Table 3.

In order to identify patterns that could be correlated to dietary habits, bands present with a frequency of at least 20% (in at least 10 participants with the same diet) were searched. The results of the band analysis are shown in Fig 6A and Fig 6B. As far as the V3 region is concerned, out of a total of 28 bands, 8 were in common for the 3 diets, while the specific bands for each diet were: 1 for the vegan group, 2 for the ovo-lacto-vegetarian group and 4 for the omnivore group. Five bands were shared between the omnivores and ovo-lacto-vegetarians, 3 between the omnivores and vegans and 3 between the vegans and ovo-lacto-vegetarians. Regarding the V3 region, the bands that appeared to be associated with the dietary habits were extracted from the gel, re-amplified and identified by means of sequencing. Representative RNA-DGGE fingerprints of the V3 region are presented in Fig 6C, while the results of band sequencing are shown in Table 2. The omnivore group showed the presence of bands identified as *Bacteroides salanitronis* and *B*. *coprocola* (bands 1 and 5, respectively) as well as *Prevotella copri* (band 7). One band was similar to the 16S rRNA gene sequences reported for an Uncultured bacterium (band 3). *Prevotella micans* and *Bacteroides vulgatus* were the specific bands for the vegetarian group (bands 2 and 4, respectively), while *Bacteroides salyersiae* (band 6) was the characteristic band for the vegan group.

**Table 2:** Microbial species identification after band sequencing of the variable V3 region of the 16S rRNA gene. Dietary habit-specific bands were selected and subjected to identification.

Band^§^	Closest relative	Closest relative Accession No.	Identity (%)
1	*Bacteroides salanitronis*	NR074616	98
2	*Prevotella micans*	NR113112	91
3	Uncultured bacterium	LN681348	100
4	*Bacteroides vulgatus*	NR074515	97
5	*Bacteroides coprocola*	NR041278	100
6	*Bacteroides salyersiae*	NR112942	96
7	*Prevotella copri*	NR113411	93

^**§**^The bands are indicated in Fig 6, Plot C.

For the band analysis of the V9 region (Fig 6B), it emerged that 8 out of a total of 19 bands were shared by the 3 diets, and the diet-specific ones were: 4 for the vegan group, 2 for the ovo-lacto-vegetarian group and 1 for the omnivore group. Two bands were shared between the omnivores and ovo-lacto-vegetarians, 1 between the omnivores and vegans and 1 was shared between the vegans and ovo-lacto-vegetarians. The RNA-DGGE fingerprints of the V9 region are presented in Fig 6D, while the band sequencing results are shown in Table 3. A specific band was identified for the vegan group, that is *Veillonella parvula* (band 5), while three bands were identified as Uncultured bacterium (bands 3, 4 and 6). The vegetarian group was characterized by the presence of *Faecalibacterium prausnitzii* (band 7) and Uncultured bacterium (band 2), while *Escherichia coli* (band 1) characterized the omnivore group.

**Table 3:** Microbial species identification after band sequencing of the variable V9 region of the 16S rRNA gene. Dietary habit-specific bands were selected and subjected to identification.

Band^§^	Closest relative	Closest relative Accession No.	Identity (%)
1	*Escherichia coli*	KP233465	99
2	Uncultured bacterium	AB247483	100
3,4	Uncultured bacterium	JQ004868	100
5	*Veillonella parvula*	NR074980	87
6	Uncultured bacterium	DQ856519	100
7	*Faecalibacterium prausnitzii*	KJ957856	100

^**§**^The bands are indicated in Fig 6, Plot D.

## Discussion

In this study, differences in fecal microbial composition, due to long-term dietary habits, have been investigated using classical plate counts and genetic fingerprinting techniques. It has frequently been reported that diet can influence gut microbiota. However, most studies have used a limited number of volunteers and have revealed only limited differences between the dietary habits. A few differences have recently been found between the gut microbiota of a small-size cohort of omnivore and vegan individuals in the USA [34].

Three large cohorts (51 subjects for each diet) with equal numbers of omnivores, ovo-lacto-vegetarians and vegans (age 30–50 years, male/female ratio ca. 1:1) have been employed for this study.

It was observed that the fecal microbiota of the ovo-lacto-vegetarian and vegan volunteers showed significantly lower microbial counts of the *Bacteroides fragilis* group. It has often been reported that long-term diets, associated with low levels of protein and animal fat intake, can decrease the levels of the *Bacteroides* genera [11]. A study on gut bacteria in children from Burkina Faso [8], who followed an agrarian diet, has shown a significant enrichment in Bacteroidetes compared to European children. The authors speculate that the abundance of this group is a consequence of the higher fiber intake, and this link has been further confirmed in larger cohort studies [35].

The mesophilic/thermophilic LAB loads (on MRS agar) were also low in the vegan and ovo-lacto-vegetarian groups, in agreement with the findings of Zimmer et al. [35]. A simple explanation for this could be the absence of food containing LAB, such as yogurt, cheese and fermented meat products, in the vegan diet, as these foods can enrich gut LAB populations since these bacteria can easily survive gastric transit [14]. A few and, in some cases, controversial *in vivo* studies have pointed out a direct but causal relationship between microbes conveyed by fermented foods and gut microbiota. The consumption of raw milk cheese was given as one of the reasons [36]. During a 42 day post-antibiotic treatment, the levels of amoxicillin-resistant intestinal enterococci fell significantly in volunteers who consumed experimental raw milk cheeses instead of pasteurized hard cheeses. This positive and, probably, transitory effect was due to the higher level of mesophilic lactobacilli, propionibacteria and enterococci in the raw milk cheeses. David et al. [9] found that *Pediococcus acidilactici* and *Staphylococcus* taxa were high in omnivore subjects, due of the consumption of cured meats in animal-based diets, which are produced with starter cultures belonging to those species.

No significant difference between the ovo-lacto-vegetarian and omnivore groups was observed in relation to the *Bifidobacterium* load, although it was significantly lower in the vegan group. However, in a recent study it has been reported that there was no significant difference for *Bifidobacterium* between the three diets [35].

Few data are available regarding the significant reduction of Proteobacteria in vegans compared to ovo-lacto-vegetarians and omnivores. In addition, Proteobacteria prefer proteins as the main source of energy, which explains their higher counts in ovo-lacto-vegetarians and omnivores. This evidence has also been reported by De Filippo et al. [8], who found that proteobacteria were present much less in a polysaccharide rich diet than in an animal protein diet. Prevotellacae counts have shown high loads in vegan and ovo-lacto-vegetarian diets, and it has been observed that Prevotella is sensitive to long-term fiber intake [8,11].

It is widely recognized that DGGE profiles can describe microbial composition and diversity, as well as shifts within the community, but they do not give information on the abundance and concentration of separate bacterial species [37]. For this reason, this limitation has partially been addressed here by conducting plate counts on several media. In addition, few studies have used RNA as a molecular marker to evaluate the metabolically active gut populations [29]. In this study, both DNA and RNA based DGGE fingerprints have been used and, through PLS-DA analysis, an impressive separation of the samples has been observed on the basis of the analyzed nucleic acid. Although the DNA fingerprints did not cluster according to the dietary types (data not shown), in agreement with a previous study [24], an approximate separation between the three diets was observed using RNA fingerprints. As a result, an analysis of the viable microbiota can contribute to the definition of the diversity of the gut environment. The specific detection of mRNA or rRNA can be attempted to target viable bacteria, even though these molecules remain available for detection after bacterial death for some generally not predictable time [27], especially for rRNA.

In addition to bacteria amplification, efforts have also been made to study fecal mycobiota, but only in 22.79% and 8.33% of the samples were DNA and cDNA obtained successfully (data not shown). These figures suggest that only a small number of yeast cells were present in the fecal samples employed in this study.

The samples were clearly separated in relation to the geographical site, and it was possible to find differences between samples from Bari (South Italy) and Parma (North Italy). This result was confirmed from an examination of the Shannon-Wiener indices. Parma had/showed the lowest diversity and Bari the highest. During the recruitment process, all the volunteers were selected in such a way as to obtain the same average age, BMI and male:female ratio in all the recruitment sites, and as a consequence, the obtained results suggest that the decrease/increase in microbiota diversity may be due to differences in food, lifestyle and/or environment. It has recently been reported that communities from Russian regions displayed similarities within the bacterial taxa associated with the gut in each region [7]. Moreover, in another study [38], substantial differences in the microbiota of healthy children and adults from Venezuela, Malawi and US metropolitan areas have been reported, although geographic differences cannot be excluded as being of influence.

In addition, it was found that only a few of the bands in the DGGE profiles were different in the three studied dietary habits, and this result is in agreement with a recent report in which only 8 bands were determined to be different for dietary habits [4]. This evidence has also been found in this study, where a very few bands of both the V3 and V9 16S rRNA gene regions were characteristic for/of each dietary habit, with some of them being common to all the diets (Fig 6). The identification of dietary habit-specific bands showed the presence of members of the *B*. *fragilis* group in the omnivore samples (*B*. *salanitronis* and *B*. *coprocola*) and this result was in good agreement with the outcome from the plate counts. *Prevotella copri* was also found as a characteristic band of the omnivore subjects, and this evidence is in good agreement with the fact that it has recently been identified by DGGE in non-agrarian diets [25]. The ovo-lacto-vegetarian group was characterized by the presence of *P*. *micans*, *B*. *vulgatus* and *Faecalibacterium prausnitzii*. In particular, *B*. *vulgatus* is recognized as being a species that is able to encode the largest number of enzymes which target the degradation of pectin, associated with an agrarian diet [39]. *Faecalibacterium prausnitzii* was found to be a characteristic species in the fecal samples of the subjects who followed a vegetarian diet [24], and it has recently been shown that it has the probiotic ability to produce vitamin B12 and to hydrolyze lactulose and galacto-oligosaccharides [40]. *Faecalibacterium prausnitzii* has also been recognized as being one of the most abundant butyrate producers in human feces. It has frequently been reported that short chain fatty acids are produced by intestinal microbiota during the fermentation of undigested polysaccharides. *B*. *salyersiae* and *Veillonella parvula* were the characteristic bands for the vegan groups, although the other sequences retrieved for this group showed greater similarity with uncultured bacteria, and this could be related to the difficulty of band isolation, which can result in too short sequences being retrieved to provide a robust phylogenetic analysis.

## Conclusion

The results presented in this study are based on a large sample size (153 healthy subjects) from 4 different Italian locations. Excluding the obvious individual variability of the volunteers, differences in the fecal microbiota, and specifically in the viable populations, have been observed. The geographical area has been shown to influence the structure of gut microbiota, probably due to the different types of foods that have been consumed. Moreover, the abundance of the *B*. *fragilis* group in omnivores has been confirmed as well as several differences between vegans and ovo-lacto-vegetarians on the LAB load by means of plate counts and band identification. Overall, these findings confirm that, type of food consumed more that the dietary habits or geographical origin, can have an impact on fecal microbiota.

## Supporting Information

S1 Fig PLS-DA models based on viable counts. PLS-DA models based on similarity matrix data from plate counts built as a function of the diet: omnivore (red), ovo-lacto-vegetarian (green) and vegan (blue).
(TIF)

S2 Fig Dendrogram of similarity generated from the digitized PCR-DGGE fingerprints of DNA. A combined data matrix of all the fingerprints for the V3 and V9 regions of 16S rRNA was obtained, while the dendrogram of similarity was obtained by means of the unweighted pair group method using an/the arithmetic average (UPGMA) clustering algorithm. Diet, sex, geographical site and sample codes are also reported.
(TIF)

S3 Fig Dendrogram of similarity generated from the digitized PCR-DGGE fingerprints of RNA. A combined data matrix of all the fingerprints for the V3 and V9 regions of 16S rRNA was obtained, while the dendrogram of similarity was obtained by means of the unweighted pair group method using an/the arithmetic average (UPGMA) clustering algorithm. Diet, sex, geographical site and sample codes are also reported.
(TIF)

S4 Fig PLS-DA models built on the similarity distance matrix on RNA-DGGE similarity matrix from each geographical site group using the diet as the discriminating factor. The samples are color coded as a function of the diet: omnivore (red), ovo-lacto-vegetarian (green) and vegan (blue).
(TIF)
