# Supplementary figures and images for: Fecal Microbiota in Healthy Subjects Following Omnivore, Vegetarian and Vegan Diets: Culturable Populations and rRNA DGGE Profiling
Source: PLoS One. 2015 Jun 2;10(6):e0128669. doi: 10.1371/journal.pone.0128669 (PMC4452701; doi:10.1371/journal.pone.0128669)

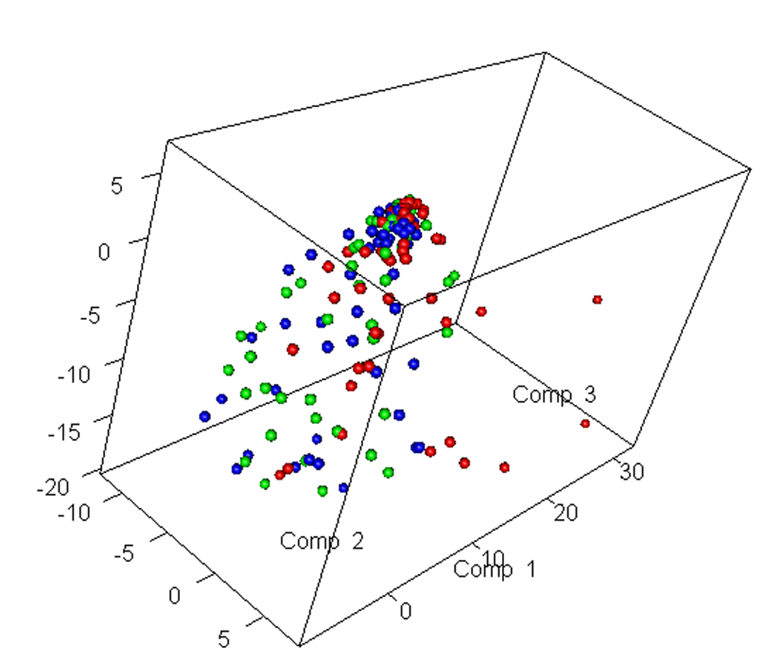

Supplement: S1 Fig — PLS-DA models based on similarity matrix data from plate counts built as a function of the diet: omnivore (red), ovo-lacto-vegetarian (green) and vegan (blue). (TIF) [file pone.0128669.s001.tif]

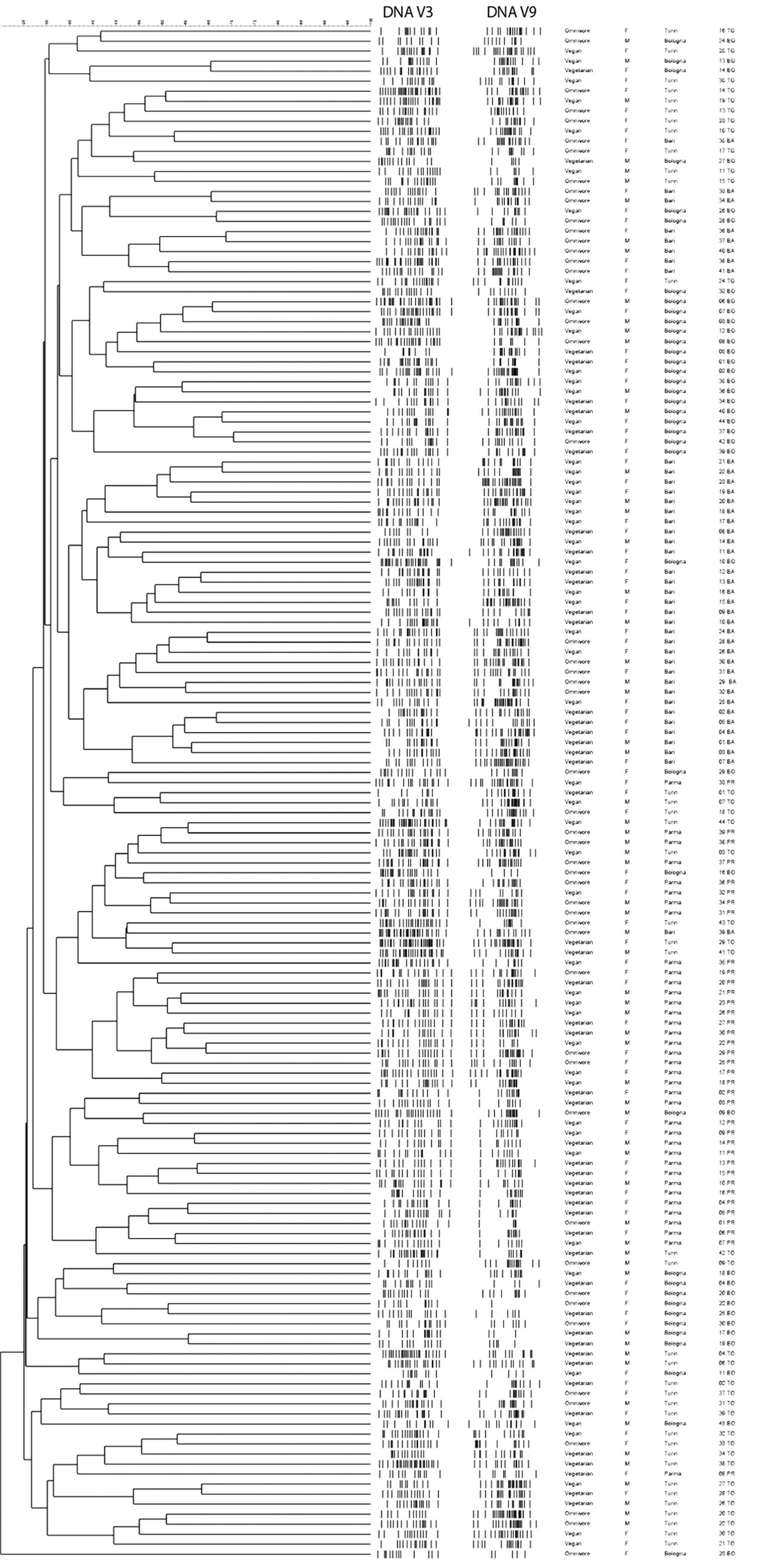

Supplement: S2 Fig — A combined data matrix of all the fingerprints for the V3 and V9 regions of 16S rRNA was obtained, while the dendrogram of similarity was obtained by means of the unweighted pair group method using an/the arithmetic average (UPGMA) clustering algorithm. Diet, sex, geographical site and sample codes are also reported. (TIF) [file pone.0128669.s002.tif]

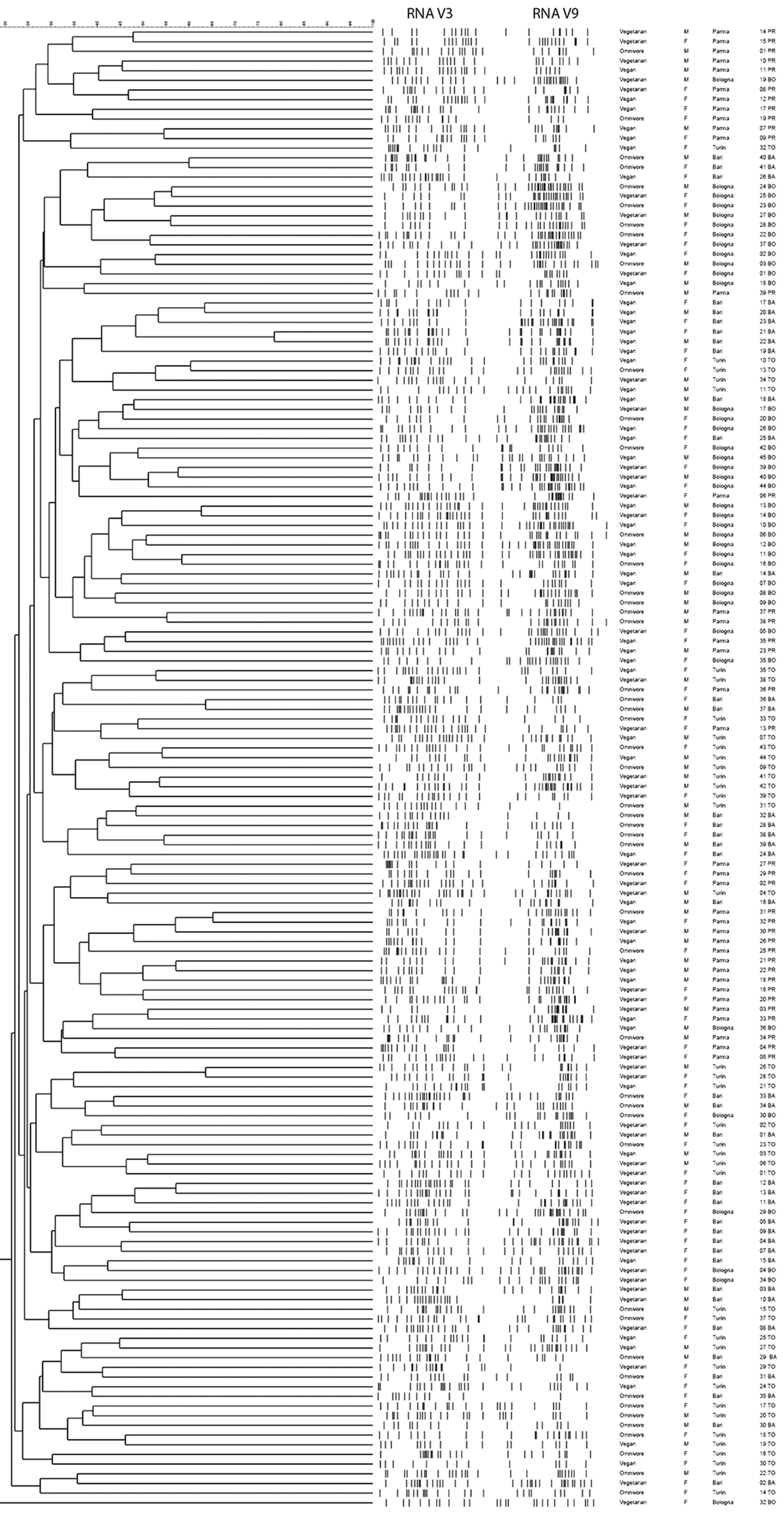

Supplement: S3 Fig — A combined data matrix of all the fingerprints for the V3 and V9 regions of 16S rRNA was obtained, while the dendrogram of similarity was obtained by means of the unweighted pair group method using an/the arithmetic average (UPGMA) clustering algorithm. Diet, sex, geographical site and sample codes are also reported. (TIF) [file pone.0128669.s003.tif]

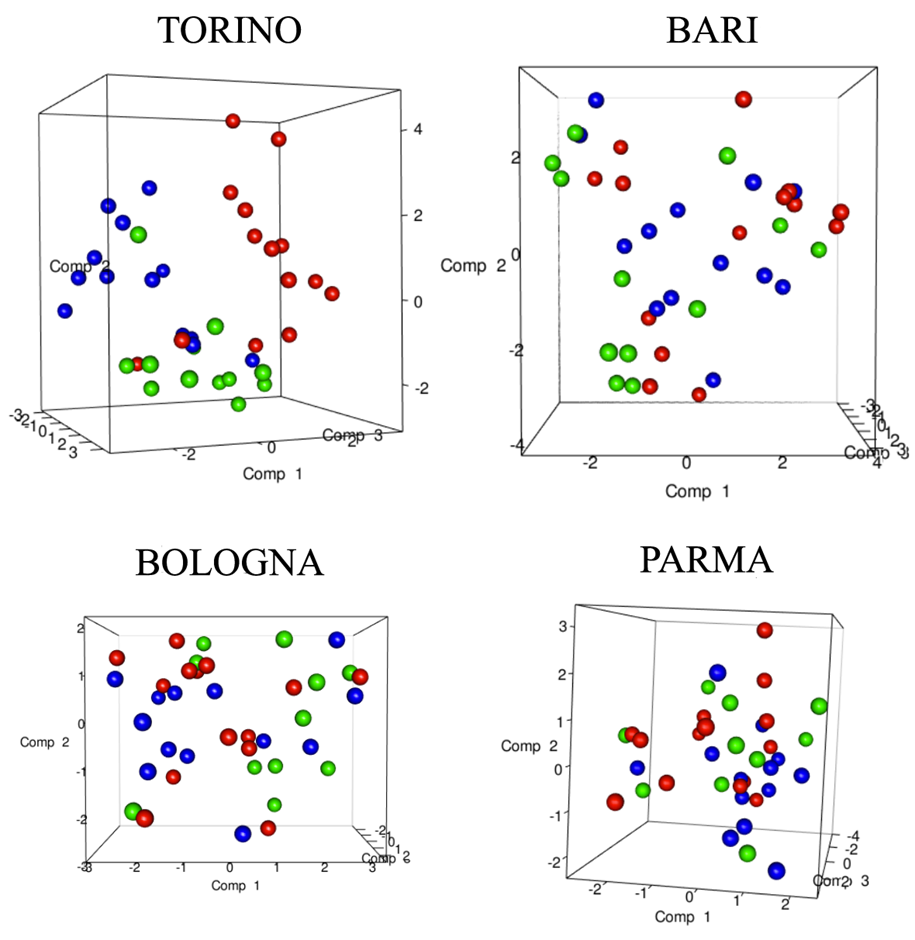

Supplement: S4 Fig — The samples are color coded as a function of the diet: omnivore (red), ovo-lacto-vegetarian (green) and vegan (blue). (TIF) [file pone.0128669.s004.tif]
